# Bacteria and Their Products
*Bacteria and their Products. By German Sims Woodhead, M.D. Edin.) Walter Scott: Contemporary Science Series. 1891.


**Published:** 1891-08-08

**Authors:** 


### PRACTITIONERS' BOOKSHELF.

### BACTERIA AND THEIR PRODUCTS.*

This little work does not attempt to compete with our larger text-books on the same subject, but, as a popular and philosophical rendering of what is usually regarded as a decidedly dry science, it is a most useful addition to our literature, and cannot fail to commend itself to all classes of readers. By happy comparison and analogy the important part which micro-organisms play in the economy of nature is brought home to us in a very vivid and interesting manner. It is to be regretted, however, that the illustrations are so poor that they appear almost anachronisms when compared with the excellent results attainable by photo-mechanical processes; and it is a pity, too, that from want of completeness the appendix just misses being a really useful guide to the practical student.

* Bacteria and their Products. By German Sims Woodhead, M.D. Edin.) Walter Scott: Contemporary Science Series. 1891.

